# Enhanced Marine Biodegradation of Polycaprolactone through Incorporation of Mucus Bubble Powder from Violet Sea Snail as Protein Fillers

**DOI:** 10.3390/polym16131830

**Published:** 2024-06-27

**Authors:** Koh Yoshida, Sayaka Teramoto, Jin Gong, Yutaka Kobayashi, Hiroshi Ito

**Affiliations:** 1 Department of Organic Materials Science, Graduate School of Organic Materials Science, Yamagata University, 4-3-16 Jonan, Yonezawa 992-8510, Yamagata, Japan; t241691d@st.yamagata-u.ac.jp (K.Y.); kobayashi.y@yz.yamagata-u.ac.jp (Y.K.); 2 Aquaculture Division, Iwate Fisheries Technology Center, 3-75-3 Heita, Kamaishi 026-0001, Iwate, Japan; mochi.saya@gmail.com

**Keywords:** marine biodegradation, biochemical oxygen demand (BOD) test, polycaprolactone (PCL), protein filler, mucus bubble, violet sea snail (Janthina globosa), biocomposite

## Abstract

Microplastics’ spreading in the ocean is currently causing significant damage to organisms and ecosystems around the world. To address this oceanic issue, there is a current focus on marine degradable plastics. Polycaprolactone (PCL) is a marine degradable plastic that is attracting attention. To further improve the biodegradability of PCL, we selected a completely new protein that has not been used before as a functional filler to incorporate it into PCL, aiming to develop an environmentally friendly biocomposite material. This novel protein is derived from the mucus bubbles of the violet sea snail (VSS, Janthina globosa), which is a strong bio-derived material that is 100% degradable in the sea environment by microorganisms. Two types of PCL/bubble composites, PCL/b1 and PCL/b5, were prepared with mass ratios of PCL to bubble powder of 99:1 and 95:5, respectively. We investigated the thermal properties, mechanical properties, biodegradability, surface structure, and crystal structure of the developed PCL/bubble composites. The maximum biochemical oxygen demand (BOD) degradation for PCL/b5 reached 96%, 1.74 times that of pure PCL (≈55%), clearly indicating that the addition of protein fillers significantly enhanced the biodegradability of PCL. The surface morphology observation results through scanning electron microscopy (SEM) definitely confirmed the occurrence of degradation, and it was found that PCL/b5 underwent more significant degradation compared to pure PCL. The water contact angle measurement results exhibited that all sheets were hydrophobic (water contact angle > 90°) before the BOD test and showed the changes in surface structure after the BOD test due to the newly generated indentations on the surface, which led to an increase in surface toughness and, consequently, an increase in surface hydrophobility. A crystal structure analysis by wide-angle X-ray scattering (WAXS) discovered that the amorphous regions were decomposed first during the BOD test, and more amorphous regions were decomposed in PCL/b5 than in PCL, owing to the addition of the bubble protein fillers from the VSS. The differential scanning calorimeter (DSC) and thermal gravimetric analysis (TGA) results suggested that the addition of mucus bubble protein fillers had only a slight impact on the thermal properties of PCL. In terms of mechanical properties, compared to pure PCL, the mucus-bubble-filler-added composites PCL/b1 and PCL/b5 exhibited slightly decreased values. Although the biodegradability of PCL was significantly improved by adding the protein fillers from mucus bubbles of the VSS, enhancing the mechanical properties at the same time poses the next challenging issue.

## 1. Introduction

The environmental pollution of plastics has been primarily a land-based concern for a long time, with little attention paid to the problem in the ocean [1]. However, nowadays, from approximately 4.8 to 12.7 million tons of plastic are dumped into the world’s oceans each year [2]. These microplastics spread throughout the ocean and do not disappear for hundreds of years, causing major damage to organisms and ecosystems [3]. In response to this problem in the ocean, marine degradable plastics are currently the focus of attention [4]. Among them, polycaprolactone (PCL) has attracted attention in recent years because of its advantages such as a high biocompatibility, high biodegradability, and high electrospinning properties, which are applied in a wide range of fields such as biomedical, tissue engineering, and medical implants. Its crystallization temperature is about 60 °C and its melting point is 59–64 °C, making it easy to prepare and mold at a low temperature [5]. The properties of PCL can be improved by blending it with other fibers and polymers to achieve better properties [6,7,8,9], and the addition of fillers impacts the mechanical and thermal properties of PCL, especially in the case of biocomposites [10,11,12]. PCL biocomposite materials have been reported by researchers, but the fillers they used were not marine-derived proteins. Instead, they utilized agricultural waste such as wheat bran and cellulose nanofibers. Additionally, their primary focus was to improve the mechanical properties and water absorption properties of PCL [13,14,15]. Based on our search, there are no reports on incorporating marine-derived proteins as functional fillers into PCL with the objective of enhancing its biodegradability.

To further enhance the biodegradability of PCL, our approach is to incorporate the protein fillers from mucus bubbles of the violet sea snail (VSS) into PCL. The VSS we used in this work was Janthina globosa, Swainson, 1822, a marine mollusk, a species of the genus Janthina in the family Epitoniidae (superfamily Epitonioidea). They float on the surface of the open ocean suspended under a float constructed from bubbles of air coated with mucus [16]. All species of VSSs are cosmopolitan, living in temperate and tropical seas and swarming. We selected mucus bubbles from VSS as protein fillers for two main reasons. Firstly, VSS is a marine animal, and its mucus bubbles are marine proteins that are expected to be fully decomposed in the sea environment by microorganisms. Secondly, the mucus bubbles from VSSs are strong enough to support VSSs’ survival at sea. Therefore, this strong bio-derived protein material attracted us to use it as a filler to enhance the marine biodegradation of PCL. Our objective was to develop an environmentally friendly and functional biocomposite material through the incorporation of mucus bubble protein fillers into PCL to create PCL/bubble composites. Two types of PCL/bubble composites, PCL/b1 and PCL/b5, were prepared with mass ratios of PCL to bubble powder of 99:1 and 95:5, respectively. The thermal properties, mechanical properties, biodegradability, surface structure, and crystal structure of the developed PCL/bubble composites were investigated.

## 2. Materials and Methods

### 2.1. Preparation of PCL/Mucus Bubble Powder (PCL/Bubble) Sheets

#### 2.1.1. Preparation of Dried Mucus Bubbles from the VSS

The snail specimens (Species: Janthina globosa, Swainson, 1822 (Mollusca: Gastropoda: Epitoniidae); Collection place: Uehara Taketomi-cho, Yaeyama-gun, Okinawa, Japan, 24°23′43.4″ N 123°51′38.7″ E) were washed with water, then the mucus bubbles were separated from the body and the egg and vacuum dried (degree of vacuum −100 kPa) at room temperature for 24 h to prepare dried mucus bubble samples.

#### 2.1.2. Preparation of Solvent-Cast Film-Shaped PCL Specimens

PCL pallets with an average molecular weight (*M_n_*) of 8000 were purchased from Sigma-Aldrich, St. Louis, MO, USA. However, because pallet-shaped PCL cannot be ground into powder even when using freeze pulverization, film-shaped PCL specimens with a thickness of approximately 2 mm were prepared following the procedure below: 5 g of PCL was dissolved in 40 mL of tetrahydrofuran (THF). The mixture was then stirred at room temperature for 24 h and left undisturbed for 24 h. Subsequently, the PCL films were obtained and transferred to a vacuum dryer, where they were vacuum dried at room temperature for 24 h (degree of vacuum −100 kPa) to completely remove the THF solvent.

#### 2.1.3. Preparation of PCL/Bubble Composite Powder via Freeze Grinding

The prepared dried mucus bubbles and film-shaped PCL were placed together in the grinding jar and ground at 25 Hz for 2 min using a cryo milling machine (CryoMill, Retsch, Verder Scientific Co., Ltd., Haan, Germany). Two types of PCL/bubble composite powders were prepared with mass ratios of PCL to bubble of 99:1 (PCL/b1) and 95:5 (PCL/b5). Pure PCL 100% (PCL) and mucus bubble powder were also prepared in the same manner as comparative specimens.

#### 2.1.4. Preparation of PCL/Bubble Composite Sheets by Heat Pressing

PCL/b1, PCL/b5, and pure PCL sheets were prepared by heat pressing their respective powders prepared above. Hot pressing was conducted on a vacuum heat press machine (1824, Imoto Machinery Co., Ltd., Kyoto, Japan) at 70 °C for 5 min under a pressure of 10 MPa, followed by cooling pressing using a cooling press machine (MP-WC, Toyo Seiki, Ltd., Tokyo, Japan) at 20 °C for 5 min under a pressure of 0.5 MPa. The resulting hot-pressed sheets had q thickness ranging from 250 to 380 μm and were used for biodegradation, mechanical properties, and other testing.

### 2.2. Characterization

#### 2.2.1. Differential Scanning Calorimetry (DSC)

The melting and crystallization behavior of the PCL/bubble composites and PCL were studied on a DSC measuring machine (M-DSC Q-2000, TA Instruments, New Castle, DE, USA) operating under a nitrogen flow. Samples of about 5 mg were heated from 20 °C to 150 °C at a heating rate of 10 °C/min, held for 1 min, then cooled to −50 °C at a cooling rate of 5 °C/min and held for 1 min, and finally reheated again to 150 °C at a rate of 10 °C/min.

#### 2.2.2. Thermal Gravimetric Analysis (TGA)

The thermal stability of the PCL/bubble composites, PCL, and mucus bubbles from the VSS was studied using a TGA (Q-50, TA Instruments, New Castle, DE, USA) at a heating rate of 10 °C/min from room temperature to 700 °C in a nitrogen atmosphere. The sample weight was more than 10 mg.

#### 2.2.3. Tensile Testing

The mechanical properties of the specimens were determined by tensile tests using a versatile tensile testing machine (AGX-X, SHIMADZU, Kyoto, Japan). The specimens were dumbbell-shaped, punched out from hot-pressed 250–380 μm thick films, with dimensions of 35 mm × 6 mm × 2 mm (test specimen No. 7, JIS K6251 standard [17]). The specimens were elongated at a constant rate of 20 mm/min until the specimens broke at room temperature. The sheets of PCL/b1, PCL/b5, and PCL were measured. The tensile strength at break and yield, elongation at break, Young’s modulus, and toughness were evaluated from the tensile stress–strain curves. Testing was run on a minimum of five specimens. The reported data were the average of the results of five specimens.

#### 2.2.4. Wide-Angle X-ray Scattering (WAXS)

To determine the degree of crystallinity ($X_{c}$), WAXS measurements were conducted on an X-ray diffractometer (Ultima IV, Rigaku, Tokyo, Japan). The sheets of PCL/b1, PCL/b5, and PCL were measured before and after biodegradation testing. The radiation source was nickel-filtered Cu Kα radiation with a wavelength of 1.5418 Å. The samples were scanned from 3° to 50° at a scanning rate of 2°/min. The $X_{c}$ was calculated using Formula (1).

(1) $$X_{c}=\frac{I_{c}}{I_{c}+I_{a}}\times 100$$

where *I_c_* is the scattering intensity of the crystalline region and *I_a_* is the scattering intensity of the amorphous region determined by the waveform separation.

#### 2.2.5. Biodegradability Testing

##### Biochemical Oxygen Demand (BOD)

The BOD biodegradation was evaluated with a BOD instrumentation setup consisting of an OxiTop IDS A12 measuring head and a 250 mL BOD reactor (Central Scientific Corporation, Tokyo, Japan). To enhance the initial activity, extracted seawater containing an increasing number and diversity of bacteria was prepared by mixing 100 g of sediment with 600 mL of raw seawater, followed by stirring and sonication. The resulting mixture was then added to the BOD reactor as an inoculum. The sample was placed into the BOD reactor containing the extracted seawater, and the OxiTop IDS A12 measuring head was attached to the head of a BOD reactor. The biodegradability testing was conducted at 20 °C for 56 days. To sustain the microbial activity, two nutrient solutions, 0.5 g/L of NH_4_CL as a nitrogen source (solution N) and 0.1 g/L of KH_2_(PO_4_) as a phosphorus source (solution P), were added into the extracted seawater twice (after 14 days and after 28 days). Each time, 3.0 mL of solution N and 3.0 mL of solution P were added to 300 mL of extracted seawater. In this study, we discussed the BOD biodegradation of PCL/b5 sheets and PCL sheets. The BOD biodegradability (%) was defined with the following Formula (2).

(2) $$BOD\;biodegradability\;(\%)=\frac{{BOD}_{sample}-{BOD}_{blank}\;}{ThOD}\times 100$$

Here, ${BOD}_{sample}$ and ${BOD}_{blank}$ are the experimentally observed values of the oxygen consumption of the sample and a blank medium, respectively. *ThOD* is a theoretically calculated value of oxygen demand of the sample, which was obtained by consuming the sample completely degraded into CO_2_ and H_2_O. Since the composition of mucus bubbles from the VSS was not clearly identified, we calculated *ThOD* by referencing the composition of jellyfish mucin (known as qniumucin) [18,19].

##### Weight Loss in Extracted Seawater

The same extracted seawater used in the BOD test was also employed for conducting biodegradation tests by measuring the weight loss of the PCL/b5 and PCL sheets. The initial weight of these samples was measured using a precision electronic scale, then these samples were wrapped in PE mesh, soaked in 250 mL of the same extracted seawater, and then incubated in a constant temperature and humidity chamber (KCL-2000W, EYELA, Tokyo, Japan) at 20 °C with a humidity level of 50% RH. After a 56-day incubation period, the samples were washed twice with distilled water, air dried, and their final weights were measured following the same procedure as the initial measurements. The degradation was estimated by calculating the percentage of weight loss. All experiments were performed in triplicate.

#### 2.2.6. Scanning Electron Microscope (SEM)

The assessment of bio-erosion on the surfaces of the PCL/b5 and PCL sheets, both before and after the BOD biodegradability testing, was visualized with a SEM (Miniscope TM-1000, Hitachi High-Technologies Corporation, Tokyo, Japan) operated at accelerating voltages of 10–15 kV. The samples were sputter-coated with platinum-palladium for SEM.

#### 2.2.7. Contact Angle Measurement

To detect changes in surface hydrophilicity, contact angle measurements were carried out with a drop shape analyzer system (DSA-25, KRÜSS, Hamburg, Germany) for the PCL/b5 and PCL sheets before and after the BOD tests. Water was used as the test liquid, and contact angles were measured in a state of equilibrium with a minimum of three replicates for each sample.

## 3. Results and Discussion

### 3.1. Thermal Properties of PCL/Bubble Composite Sheets and Powder

**Figure 1:**
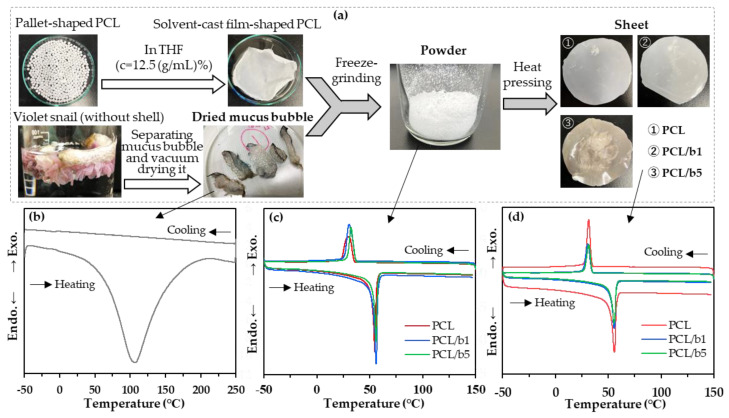
The preparation procedure of sheets and powder for pure PCL (PCL) and PCL/bubble composites (PCL/b1 and PCL/b5) (**a**), and the DSC curves of dried mucus bubbles from the VSS (**b**), the powder (**c**), and the sheet (**d**) samples of PCL, PCL/b1, and PCL/b5.

The sheets and powder for the PCL/bubble composites (PCL/b1 and PCL/b5) and pure PCL (PCL) were prepared according to the procedure shown in Figure 1a. The DSC curves of dried mucus bubbles from the VSS (Figure 1b), the powder (Figure 1c), and the sheet (Figure 1d) samples of PCL, PCL/b1, and PCL/b5 are also shown in Figure 1. The crystallization temperature (*T*_c_) and melting point (*T*_m_) for these samples measured by DSC are summarized and listed in Table 1.

The dried mucus bubbles from the VSS showed a broad melting peak with a peak value of 108.9 °C, but the *T*_c_ was not observed during the cooling process. For all powder and sheet samples of PCL, PCL/b1, and PCL/b5, a clear single exothermal peak near 31 °C and a clear single endothermal peak near 55 °C were observed, which belongs to the *T*_c_ and *T*_m_ of PCL crystallites, respectively. No significant changes in either *T*_c_ or *T*_m_ were observed between PCL/bubble composites and pure PCL, which indicates that the addition of mucus bubble powder only had a slight impact on the *T*_c_ and *T*_m_ of PCL.

**Figure 2:**
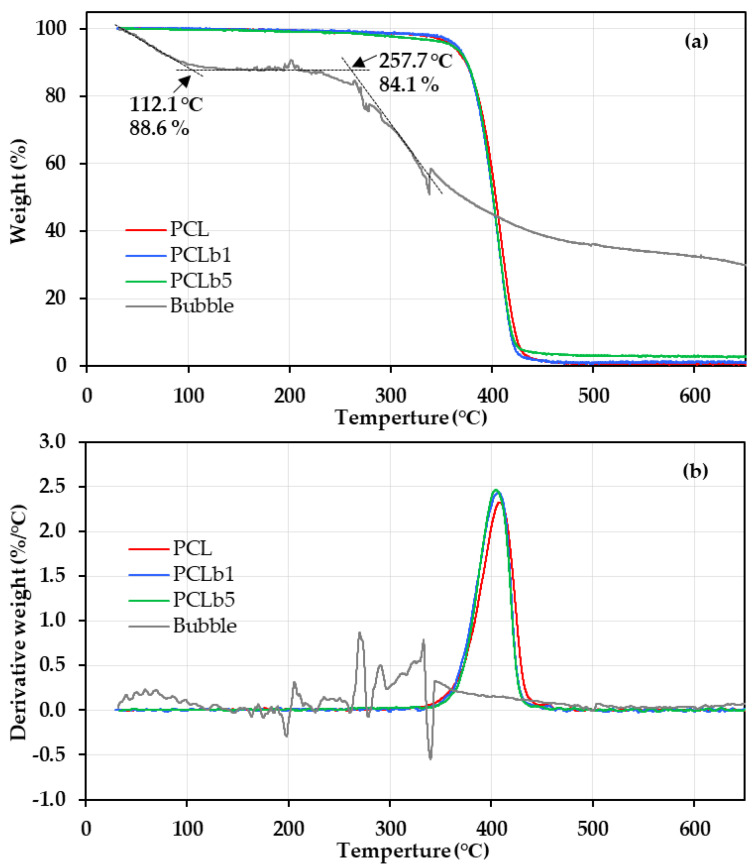
The weight loss vs. temperature plots (**a**) and their corresponding derivative curves (**b**) obtained from TGA measurements for PCL, PCL/b1, PCL/b5, and mucus bubble powder from the VSS.

The thermal stabilities of PCL, PCL/b1, PCL/b5, and the violet snail mucus bubble powder were assessed through TGA under a nitrogen environment. Figure 2 presents the weight loss vs. temperature plots (a) and their corresponding derivative curves (b) obtained from TGA measurements. Detailed results are provided in Table 2.

The TGA plot (Figure 2a) revealed two temperature ranges for the weight loss of the mucus bubble. The first weight loss, below 150 °C, beginning at 112.1 °C with a loss of 88.6%, was attributed to the evaporation of water absorbed in the mucus bubble under natural storage conditions, which indicated that the mucus bubble had a moisture content of approximately 11%. The second weight loss between 200 and 500 °C was due to the thermal degradation of the mucus bubble. Consequently, the TGA results indicated that the mucus bubble exhibited a thermal degradation onset temperature (*T*_onset_) at 257.7 °C with a weight loss of approximately 4.5%.

In comparison, according to the TGA results, PCL, PCL/b1, and PCL/b5 exhibited a *T*_onset_ at around 380 °C with a total weight loss of less than 0.5%. Additionally, PCL, PCL/b1, and PCL/b5 displayed temperatures at which there were weight losses of 5 wt% (*T*_5%_) and 10 wt% (*T*_10%_) exceeding 360 °C and approximately 375 °C, respectively. These findings clearly demonstrate that the addition of mucus bubble powder minimally affected the thermal degradation behavior of PCL. In other words, the PCL composites, PCL/b1 and PCL/b5, exhibited a thermal stability level comparable to that of pure PCL.

Furthermore, the derivative peak (Figure 2b), representing the inflection point (*T*_inflep_) denoting the greatest rate of change on the weight loss curve, revealed that, although the *T*_inflep_ of the mucus bubble (276.1 °C and 292.6 °C) was lower than that of PCL, PCL/b1, and PCL/b5 (approximately 405 °C), the addition of the mucus bubble showed a minimal impact on the *T*_inflep_ of PCL as well. Overall, the TGA results suggested that the bubble-added PCL composites, PCL/b1 and PCL/b5, exhibited a high level of thermal durability comparable to that of pure PCL. These results suggest that the impact of adding mucus bubbles on thermal properties is smaller than that of other biocomposite materials using natural fibers such as cellulose and macaiba [10,11].

### 3.2. Mechanical Properties of PCL/Bubble Composite Sheets

**Figure 3:**
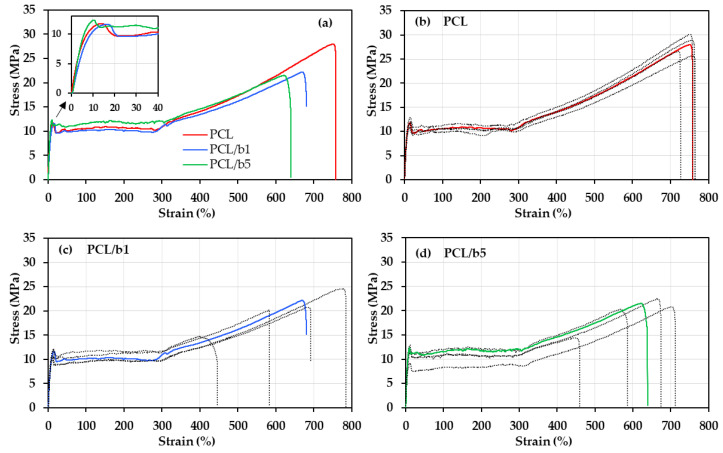
Tensile stress–strain curves for PCL, PCL/b1, and PCL/b5. (**a**) Comparison of typical curves for PCL, PCL/b1, and PCL/b5, curves for (**b**) PCL, (**c**) PCL/b1, and (**d**) PCL/b5 obtained from five specimens. The red line in (**b**), blue line in (**c**), and green line in (**d**) were selected for comparison in (**a**) as a typical curve for PCL, PCL/b1, and PCL/b5, respectively.

**Figure 4:**
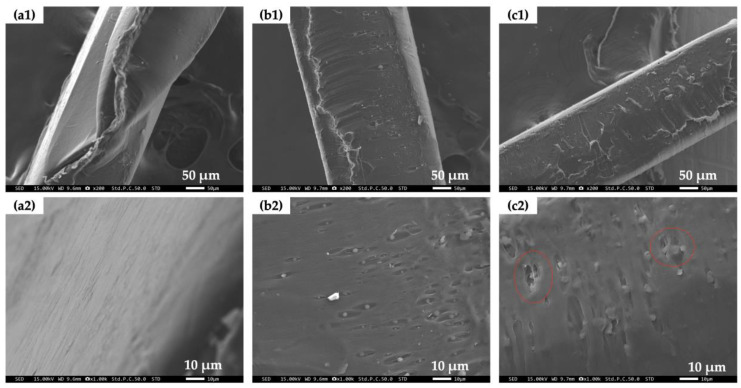
SEM micrographs of cross-sectional view for PCL (**a1**,**a2**), PCL/b1 (**b1**,**b2**), and PCL/b5 (**c1**,**c2**) sheets. (**a1**,**b1**,**c1**) are low-magnification pictures, and (**a2**,**b2**,**c2**) are relatively high-magnification pictures. Regions inside red circles in (**c2**) indicate the agglomeration of mucus bubble particles in PCL/b5.

Tensile tests were conducted to evaluate the mechanical properties of the PCL/bubble composites. The tensile stress–strain curves of PCL, PCL/b1, and PCL/b5 are presented in Figure 3. The average values of the mechanical properties are given in Table 3. In Figure 3a, the breaking strength, yield strength, breaking strain, toughness, and Young’s modulus of the PCL and bubble-added composites PCL/b1 and PCL/b5 were compared. Young’s modulus was measured in the strain range of 0.05–0.25%, where stress is proportional to strain. Compared to pure PCL, the mucus-bubble-added composites PCL/b1 and PCL/b5 exhibited a little lower breaking strength and breaking stain, and overall, their toughness decreased from 117.0 MJ/m^3^ to 85.5 MJ/m^3^. On the other hand, the yield strength of PCL/b1 and PCL/b5 increased, notably, the Young’s modulus of PCL/b5 rose to 238.0 MPa, showing a 13% increase from PCL’s 210.2 MPa. Also, compared to pure PCL (Figure 3b), variations in the physical properties for PCL/b1 (Figure 3c) and PCL/b5 (Figure 3d) were observed, suggesting that the addition of mucus bubbles also caused the generation of structural heterogeneity in the bubble-added composites. This can be verified through SEM micrographs of cross-sectional view of the sheet samples (Figure 4).

Figure 4 shows cross-sectional views of the sheet samples observed with a scanning electron microscope (SEM) and the SEM micrographs. The cross-sectional surface was relatively flat for PCL (Figure 4(a1,a2)), while, with the addition of mucus bubbles, it transformed into a porous structure with particles of mucus bubbles having a diameter of less than 5 μm inside the pores for both PCL/b1 (Figure 4(b1,b2)) and PCL/b5 (Figure 4(c1,c2)). We also observed a slight increase in the agglomeration of mucus bubble particles in PCL/b5 with a higher addition amount of mucus bubbles (Figure 4(c2), regions inside red circles). The porous structure and particle agglomeration were primarily considered to contribute to the decrease in the breaking strain and the increase in the yield strength, especially for PCL/b5 composites. Other researchers have also reported similar results that the aggregation of the blended fillers or a large size of these fillers can cause stress concentrations, further resulting in the degradation of the mechanical properties [14,15,20,21,22,23].

### 3.3. Degradability of PCL/Bubble Composites in Marine Environments

**Figure 5:**
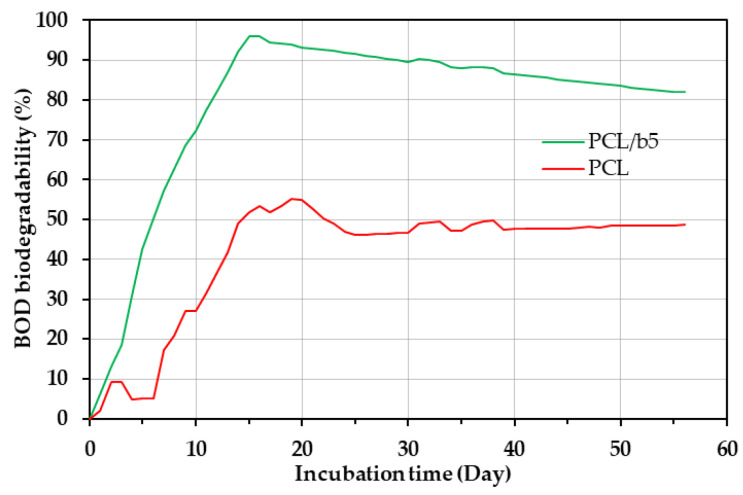
BOD test results for PCL/b5 sheets with a 95:5 mass ratio of PCL to mucus bubble and pure PCL sheets at 20 °C for 56 days.

The degradability of the PCL/bubble composite PCL/b5 sheets was evaluated by the BOD biodegradation testing with extracted seawater containing an increasing number and diversity of bacteria. Pure PCL sheets were evaluated in the same manner as the comparison. The BOD biodegradation curves are shown in Figure 5. The BOD test results indicated that the biodegradation of both PCL/b5 and pure PCL took place in two steps. Initially, within the first 15–20 days, quick degradation was observed. PCL/b5 exhibited degradation 1.51 times faster (6.9% per day) than PCL (4.6% per day). Subsequently, the degradation rates for both samples slowed down around 25 days after the initiation of the BOD testing. The degree of biodegradation peaked after approximately 15 days for PCL/b5 and around 20 days for pure PCL. The maximum BOD degradation for pure PCL was about 55%, whereas for PCL/b5, it reached 96%, which was 1.74 times that of pure PCL. These results clearly indicated that the inclusion of mucus bubble powder from the VSS in PCL/b5 resulted in a significantly higher BOD degradation compared to pure PCL, that is, the addition of protein fillers from mucus bubbles significantly enhanced the biodegradability of PCL.

### 3.4. Weight Loss of PCL/Bubble Composites

The PCL/bubble composite PCL/b5 and pure PCL sheet samples were immersed in the same extracted seawater used in the BOD test at 20 °C. After 56 days, the samples were collected, and the weight loss was calculated based on the weights of the sheets before and after the test. The weight loss for each sample and average weight loss are summarized in Table 4. PCL/b5 presented a 34.1% average weight loss, slightly higher than that of pure PCL, which was 33.4%. It was also observed that the three PCL/b5 samples showed similar weight loss values, while there was greater variations in weight loss for three pure PCL samples. This suggests that, in terms of weight loss, the biodegradation of PCL/b5 occurred slightly more easily and consistently compared to that of pure PCL.

### 3.5. Surface Structure of PCL/Bubble Composite Sheets before and after the BOD Test

**Figure 6:**
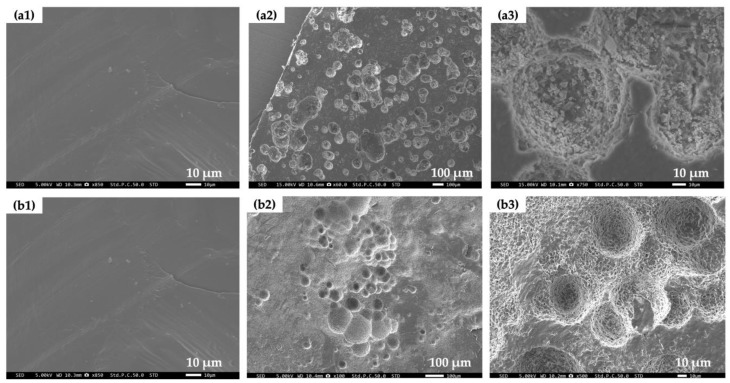
SEM images of surface of PCL/b5 (**a1**–**a3**) and PCL (**b1**–**b3**) sheets before and after BOD test. (**a1**,**b1**): before BOD test (initial sheets); (**a2**,**b2**): after the BOD test (soaked in extracted seawater at 20 °C for 56 days); and (**a3**,**b3**): relatively high-magnification pictures of (**a2**,**b2**). It is evident that the surfaces of both PCL/b5 and PCL sheets underwent significant decomposition.

The changes in surface morphology of the PCL/bubble composite (PCL/b5) sheets and pure PCL sheets before and after the BOD test were observed with SEM. The SEM images of the surfaces of the PCL/b5 and PCL sheets before and after BOD test are presented in Figure 6. For both PCL/b5 and pure PCL, compared to the smooth surface of the initial sheets (Figure 6(a1,b1)), after the BOD test, their surface became severely uneven and lots of indentations with diameters of 10–200 μm on the surface were generated (Figure 6(a2,b2)). According to the relatively high-magnification SEM images (Figure 6(a3,b3)), a large amount of fine particles (200 nm–5 μm) were detected inside the indentations for PCL/b5, while a hollow network structure resembling a bird’s nest was observed for PCL. These observation results definitely confirmed the occurrence of degradation. Additionally, it was noted that PCL/b5 underwent more significant degradation compared to pure PCL. This was evidenced by the considerable amount of fine particles observed, suggesting that PCL/b5 broke down into smaller pieces to a greater extent.

**Figure 7:**
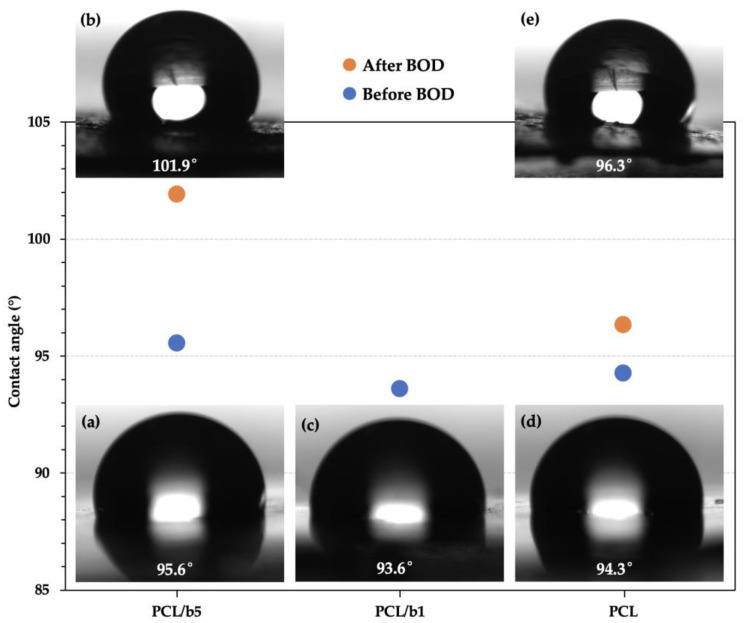
Changes in water contact angle of PCL/b5, PCL/b1, and PCL sheets before and after the BOD test. (**a**,**b**): PCL/b5; (**c**): PCL/b1; (**d,e**): PCL; (**a,c,d**): before BOD test (initial sheets); (**b,e**) after BOD test (soaked in extracted seawater at 20 °C for 56 days).

Furthermore, the changes in surface structure were also investigated by measuring the water contact angle to determine whether the hydrophobicity of the PCL/b5 and pure PCL sheets changed before and after the BOD test. Figure 7 shows the results of the contact angle measurement. The average contact angles before the BOD test were 95.6° for PCL/b5, 93.6° for PCL/b1, and 94.3° for PCL, and after the BOD test, they were 101.9° for PCL/b5 and 96.3° for PCL. All sheets exhibited a water contact angle of more than 90°, indicating that all sheets were hydrophobic. It was also observed from the results that the surfaces of PCL/b5 and PCL became more hydrophobic after the BOD test. This was thought to be due to the newly generated indentations on the surface after the BOD test, which led to an increase in the surface toughness and, consequently, an increase in the surface hydrophobility, as indicated by the Wenzel roughness in Wenzel’s model [24,25,26]. 

### 3.6. Crystal Structure of PCL/Bubble Composites 

**Figure 8:**
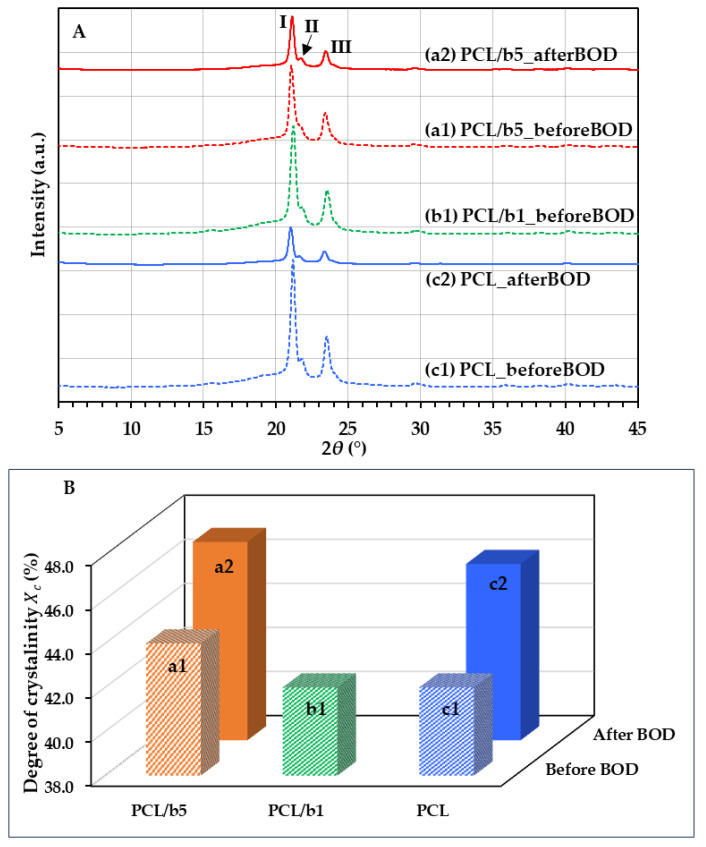
WAXS patterns (**A**) and the degree of crystallinity $X_{c}$ (**B**) of PCL/b5, PCL/b1, and PCL sheets before and after the BOD test. Three peaks (I, II and III, as shown in A) were detected for all samples.

The degradation of polyester is said to depend on the chemical composition, molecular weight, hydrophobicity/hydrophilicity, crystallinity, and surface area of the sample, etc. [27,28,29]. Aiming to discover whether crystals affected the degradability of the PCL/bubble composites, the crystal structure of the PCL/bubble composites, including PCL/b5 and PCL/b1, as well as pure PCL, was examined using WAXS at room temperature, and the degree of crystallinity ($X_{c}$) was calculated basing on the WAXS results. The sheet samples before and after the BOD test were measured. Figure 8A,B present the WAXS patterns and the $X_{c}\;$ of PCL/b5, PCL/b1, and PCL sheets before and after the BOD tests.

For all samples of PCL/b5, PCL/b1, and PCL (Figure 8A, Table 5), three peaks were observed at diffraction angles of 2θ ≈ 21.1°, 21.7° and 23.5° on the WAXS patterns, which corresponded to a *d* of around 4.2 Å, 4.1 Å, and 3.8 Å, respectively. This implied there was no obvious difference in the crystal structure with or without the addition of the mucus bubble protein fillers from the VSS, both before and after the BOD test. Regarding $X_{c}$ (Figure 8B), before the BOD test, PCL/b5 exhibited a value of 44%, higher than the 42% observed for PCL/b1 and PCL, suggesting that the addition of the bubble fillers at 5 wt% resulted in a rise in $X_{c}$. After the BOD test, both PCL/b5 and pure PCL showed a higher $X_{c}$, reaching 47% and 46%, respectively, compared to before the BOD test. In summary, the crystal structure remained largely unchanged, while $X_{c}$ increased after the BOD test. It can be concluded that the amorphous regions were decomposed first during the BOD test, and more amorphous regions were decomposed in PCL/b5 than in PCL owing to the addition of the bubble protein fillers. These protein fillers are supposed to serve as food for microorganisms, attracting them to the surrounding area and facilitating decomposition. Therefore, the addition of bubble protein fillers promoted and accelerated the biodegradation of PCL.

## 4. Conclusions

We prepared biocomposite sheets by adding protein fillers from mucus bubbles of the VSS into PCL in order to further improve the biodegradability of marine-degradable plastic PCL. The maximum BOD degradation for PCL/b5 reached 96%, 1.74 times that of pure PCL (≈55%), clearly indicating that the addition of protein fillers significantly enhanced the biodegradability of PCL. The surface morphology observation results through SEM definitely confirmed the occurrence of degradation. Compared to a hollow network structure resembling a bird’s nest observed for PCL, a large amount of fine particles ranging from 200 nm to 5 μm were detected inside the indentations for PCL/b5, suggesting that PCL/b5 underwent more significant degradation compared to pure PCL. The water contact angle measurement results exhibited that all sheets were hydrophobic (water contact angle > 90°) before the BOD test and showed the changes in surface structure after the BOD test due to the newly generated indentations on the surface, which led to an increase in surface toughness and, consequently, an increase in surface hydrophobility. The crystal structure analysis by WAXS discovered that the amorphous regions were decomposed first during the BOD test, and more amorphous regions were decomposed in PCL/b5 than in PCL owing to the addition of the bubble protein fillers. These protein fillers are supposed to serve as food for microorganisms, attracting them to the surrounding area and facilitating decomposition. Therefore, the addition of protein fillers from mucus bubbles of the VSS promoted and accelerated the biodegradation of PCL.

The DSC and TGA results suggested that the addition of mucus bubble protein fillers had only a slight impact on the thermal properties of PCL. From these results, it can be concluded that the PCL/bubble composite material was a material that had a high marine biodegradability while maintaining its thermal properties. In terms of mechanical properties, compared to pure PCL, the mucus-bubble-filler-added composites PCL/b1 and PCL/b5 exhibited a slightly lower breaking strength and breaking stain, and overall, their toughness decreased; on the other hand, their yield strength increased. The degradation of the mechanical properties was considered to be due to the aggregation of the blended fillers or the large size of the fillers. Although the biodegradability of PCL was significantly improved by adding the protein fillers from mucus bubbles of the VSS, enhancing the mechanical properties at the same time poses the next challenging issue. It is supposed that the compatibility between plastic PCL and protein fillers is not good. Firstly, conducting a detailed structure analysis of the interface between the matrix PCL and filler protein to obtain clues to further enhance the mechanical properties of PCL/bubble composites seems important. Then, proposals such as functionalizing bubble powder for better dispersion, enhancing matrix/powder interfaces, and minimizing the bubble powder size to increase the surface area would be very helpful. Furthermore, we have initiated research involving the addition of marine proteins derived from other marine organisms, such as jellyfish and hagfish, as protein controls to verify the advantage, necessity, and importance of protein fillers from VSS. We hope to share our findings on this topic in our next upcoming report.

## Figures and Tables

**Table 1:** The crystallization temperature (*T*_c_) and melting temperature (*T*_m_) of PCL, PCL/b1, PCL/b5, and mucus bubble powder from the VSS measured by DSC.

Sample			*T*_c_ (°C)	*T*_m_ (°C)
Powder	PCL		31.7	54.7
	PCL/bubble composite	PCL/b1	30.5	56.0
		PCL/b5	31.7	55.4
	Bubble		-	108.9
Sheet	PCL		31.8	55.8
	PCL/bubble composite	PCL/b1	29.5	54.0
		PCL/b5	29.3	57.1

**Table 2:** Thermal degradation temperatures obtained from TGA measurements for PCL, PCL/b1, PCL/b5, and mucus bubble powder from the VSS.

Sample			*T*_onset_ (°C)	*T*_5%_ (°C)	*T*_10%_ (°C)	*T*_inflep_ (°C)
Powder	PCL		381.5	360.3	374.1	408.8
	PCL/bubble composite	PCL/b1	378.6	364.5	375.1	406.0
		PCL/b5	381.2	360.2	375.2	404.5
	Bubble		257.7	-	-	276.1/292.6

*T*_onset_: onset temperatures, temperature at which the weight loss begins, *T*_5%_: temperature at which there is a weight loss of 5 wt%, *T*_10%_: temperature at which there is a weight loss of 10 wt%, *T*_inflep_: inflection point, temperature at which the weight loss curve exhibits the greatest rate of change.

**Table 3:** Mechanical properties of PCL, and PCL/bubble composites, including PCL/b1 and PCL/b5.

Sample	Breaking Strength (MPa)	Yield Strength (MPa)	Breaking Strain (%)	Toughness (MJ/m^3^)	Young’s Modulus (MPa)
PCL	27.5	11.2	757.2	117.0	210.2
PCL/b1	20.9	11.3	648.8	85.5	199.8
PCL/b5	20.1	12.5	626.6	85.5	238.0

**Table 4:** Values of weight loss and average weight loss for PCL/b5 and pure PCL sheets which were measured at 20 °C over a period of 56 days when they were immersed in the same extracted seawater used in the BOD test.

Sample ^(a)^		Weight Loss (%)	Average Weight Loss (%)
PCL/b5	1	34.5	34.1
	2	36.8	
	3	30.9	
PCL	1	34.6	33.4
	2	25.6	
	3	40.1	

^(a)^ Three samples (1.0 cm × 1.0 cm) were tested for each measurement. The average thickness of PCL/b5 sheets was 250 ± 20 μm and that of PCL was 230 ± 20 μm.

**Table 5:** The locations of diffraction peaks and corresponding interplanar crystal spacing *d* of PCL/b5, PCL/b1, and PCL calculated from the SAXS results.

Sample		Peak I		Peak II (Shoulder Peak)		Peak III	
		2θ (°)	*d*_1_ (Å)	2θ (°)	*d*_2_ (Å)	2θ (°)	*d*_3_ (Å)
PCL/b5	After BOD	21.14	4.20	21.65	4.10	23.45	3.79
	Before BOD	21.08	4.21	21.73	4.09	23.42	3.80
PCL/b1	Before BOD	21.21	4.19	21.81	4.07	23.55	3.78
PCL	After BOD	21.03	4.22	21.67	4.10	23.35	3.81
	Before BOD	21.20	4.19	21.83	4.07	23.54	3.78

## Data Availability

The data presented in this study are available within the article.
